# Protein Kinase C: One Pathway towards the Eradication of Latent HIV-1 Reservoirs

**DOI:** 10.1155/2012/805347

**Published:** 2012-03-05

**Authors:** Lisa N. McKernan, David Momjian, Joseph Kulkosky

**Affiliations:** Department of Biology, Chestnut Hill College, Philadelphia, PA 19118, USA

## Abstract

An effective means to eradicate latent reservoirs in HIV-1-infected individuals remains elusive. Attempts to purge these reservoirs were undertaken over a decade ago without success. The subsequent lapse in further clinical attempts since may have been justified as our knowledge of the mechanisms which underpin the latent state still evolves. Although additional novel molecular antagonists of HIV-1 latency have subsequently been reported, these candidate agents have not been tested in human trials for reservoir ablation. This review provides an overview of the protein kinase C (PKC) pathway which can be modulated by small molecular agents to induce the expression of latent HIV-1 from within infected reservoir cells. Some of these agents have been tested against select cancers with seemingly tolerable side effects. As such, modulation of the PKC pathway may yet be a viable mechanism toward HIV-1 reservoir eradication.

## 1. Introduction

Administration of highly active anti-retroviral therapy (HAART) to HIV-1-infected individuals results in effective suppression of viral replication in metabolically active cells bearing integrated viral DNA. However, a small population of infected cells is refractive to HAART treatment as a consequence of being quiescent and/or not actively expressing virus products [1–5]. This small population of cells, comprised largely of infected CD4^+^ resting T cells, constitutes the HAART-persistent latent reservoir. Most cells in this silent reservoir have long half lives [6, 7] and are hidden from immune surveillance which permits them to remain as a stable source for *de novo* viral production upon reactivation. One strategy for eradication of this reservoir rests upon the premise that cellular activation with concomitant upregulation of viral expression will hasten its elimination [8–11]. Cellular activation typically shortens the half-life of a cell relative to its quiescent counterpart, and a cell, actively expressing viral antigens, would be a more favorable target for immune clearance [12].

 Some time ago, several clinical trials were attempted to eradicate or diminish the presence of latent reservoirs using the cellular activators, OKT3 and IL-2, which primarily target T-cell responses [13–18]. These trials were ineffectual although genotypic alterations of reservoir virus in treated individuals were noted [19]. Nonetheless, these attempts clearly indicated that a broader armamentarium of agents or multiple clinical interventions were required to accomplish the elusive goal of complete reservoir eradication. The list of candidates in this armamentarium has grown to be quite lengthy. They include small hydrophobic agents within the phorbol ester family of compounds [20], as well as bryostatin-1, a macrolide lactone [21]. The phorbol ester family of compounds and select lactones modulate the protein kinase C (PKC) pathway which induces latent HIV-1 expression [20, 21]. Chromatin remodeling agents [22–26] and select cytokines, primarily the interleukins or interferons, have also been shown to upregulate viral expression from latent proviruses. [27–30].

 Clearly, compounds which upregulate latent HIV-1 expression may have clinical utility toward the eradication of HIV-1 HAART-persistent reservoirs. Their elimination would effectively represent a cure for HIV-1 infection. This paper will focus upon PKC as one pathway that may be modulated by small molecular agents toward this goal.

## 2. Overview of the Protein Kinase C Pathway

 The protein kinase C (PKC) signal cascade is comprised of, and regulated by, several isoforms [31, 32]. Each isoform exhibits select characteristics as well as varying patterns of expression in specific cell types. The PKC cascade can affect receptor upregulation or downregulation, membrane and cytoskeleton remodeling, and positive or negative regulation of transcription to mediate specific processes within the cell. These activities can have global effects on cellular functions, in particular, growth, attachment, differentiation, maturation, and death [31, 32]. These varied functions are typically mediated by PKC phosphorylation of serine and threonine residues of downstream signaling factors [33, 34]. These phosphorylated factors then serve as intermediaries in the transduction of signals to various cellular locations in order to accomplish specific effector functions.

## 3. Stimulation of the PKC Pathway

 PKC pathway activation involves the participation of the phospholipase C (PLC) superfamily of proteins for most natural cellular processes. PLCs participate in phosphatidylinositol-4,5-bisphosphate (PIP_2_) metabolism and lipid signaling pathways in a calcium-dependent manner. Similar to the PKC pathway, the PLC superfamily consists of many isoforms which differ in their mode of activation, expression levels, catalytic regulation, cellular localization, and membrane binding affinity [33]. All are capable of catalyzing the hydrolysis of PIP_2_ into two important second messenger molecules: diacylglycerol (DAG) and inositol-1,4,5-trisphosphate (IP_3_). These two second messengers have differential cellular effects. IP_3_ molecules diffuse through the cytoplasm and bind to the endoplasmic reticulum (ER) resulting in the opening of calcium channels [31–34]. The released calcium from the ER into the cytoplasm is free to bind important regulatory proteins including but not restricted to calmodulin and calcineurin. The binding of calcium to calmodulin mediates critical organismal processes such as inflammation, metabolism, apoptosis, smooth muscle contraction, intracellular movement, short-term and long-term memory, nerve growth, and the immune response [35–37].

**Figure 1:**
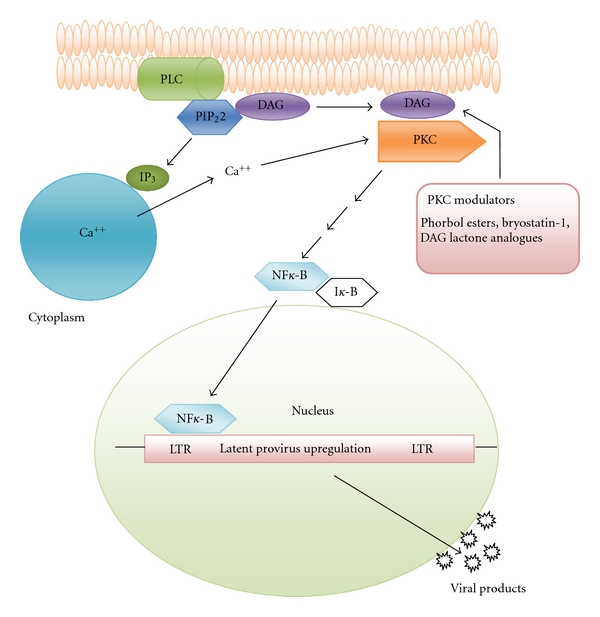
Overview of the mechanistic steps involved in PKC modulation toward the upregulation of latent HIV-1. PKC modulating agents represent functional analogues of DAG that similarly activate the PKC cascade.

 DAG, the other by-product of PLC cleavage, can activate PKC in cooperation with calcium [31–33]. The kinase activity of phosphorylated PKC then phosphorylates various protein targets and these targets in turn transduce signals broadly through select signaling pathways. These initial events are illustrated in Figure 1. Importantly, natural or synthetic phorbol esters, like phorbol-12-myristate 13-acetate (PMA), a tumor promoting agent, or prostratin, a non-tumor promoting phorbol, as well as select lactones like bryostatin-1 can serve as mimetics or analogues of DAG to modulate PKC pathway activity for the induction of latent proviral expression [20–22].

## 4. PKC Isoform Specificity and Action

All PKC isoforms consist of a regulatory domain tethered to a catalytic domain [31, 32]. There are two primary classes of PKC protein isoforms. Calcium-dependent classical PKC isoforms (cPKCs), require calcium for their activity, while calcium-independent isoforms (nPKCs) do not. The cPKCs (*α*, *β*I, *β*II, and *γ*) are calcium and DAG-dependent, whereas the novel PKCs (*δ*, *ε*, *η*, and *θ*) are calcium-independent but DAG-responsive. A third class of PCKs is referred to as atypical includes the isoforms *ξ* and *λ*/l that uniquely lack responses to calcium and DAG [31, 32, 38].

 A highly conserved cysteine-rich motif in the regulatory region of most PKC isoforms acts as the specific docking receptor for DAG as well as for phorbol esters like PMA [31]. This conserved DAG/PMA-binding domain, referred to as C1, binds two zinc ions in a zinc finger-like structure which is comprised of six cysteines and two histidines [38, 39]. The C1 domain displays a hydrophobic surface surrounding a hydrophilic cleft. Studies suggest that DAG, phorbol esters or DAG lactone binding to C1 conceals the hydrophilic cleft reducing the overt charge of this domain [38–41]. This facilitates hydrophobic interaction of PKC complexes with the cytoplasmic portion of the plasma membrane or other membranous cellular surfaces [38–41]. This translocation of PKCs in complexes with DAG or DAG analogues to the plasma membrane and other sub-cellular localizations initiates the PKC activation cascade. It is of interest that various phorbol derivatives, with differing affinities for the C1 domain of PKC isoforms, elicit different cellular responses as well as the degree to which these responses are sustained including the upregulation in expression of latent HIV-1 proviruses [20, 40].

## 5. PKC-Mediated Upregulation of Latent HIV-1 Reservoirs

 The non-tumor-promoting deoxyphorbol esters which activate PKC emerged as candidates for HIV-1 latent reservoir eradication from studies initially performed at the National Cancer Institute (NCI). NCI investigated antiviral properties of several ethnobotanical compounds including the novel phorbol ester, prostratin. Prostratin had first been identified as a constituent of the poisonous New Zealand plant *Pimelea prostrata* [42]. As observed by the ethnobotanist, Paul Cox, prostratin was later detected in bark extracts from the plant, *Homalanthus nutans,* used by native island tribesmen in Samoa as a remedy for jaundice [43]. On this basis it was speculated to have antiviral properties. Initial investigations of the purified compound by NCI revealed that prostratin inhibited infectious viral spread but readily upregulated latent HIV-1 from the quiescently infected cell lines, ACH2 and U1 [44, 45]. Prior to the introduction of HAART, there was little interest in further investigating an agent that would produce additional virus from within the cells of HIV-1-infected patients.

 The view for prostratin's utility evolved with the subsequent advent of HAART. HAART potently prevents viral spread, presumably including any virus that could subsequently be expressed from latent proviral DNA. Consequently, prostratin was proposed as a candidate for adjuvant therapy in conjunction with HAART to eradicate the latent reservoir via cellular activation without the danger of viral spread or latent reservoir reseeding [46]. Importantly, early studies clearly indicated that prostratin and other non-tumor-promoting phorbol esters, including 12-deoxyphorbol-13-phenylacetate (DPP), readily reactivated latent virus from primary HIV-1-infected patient resting T cells [20, 46–48]. This same ability has been demonstrated more recently with lactone analogues of DAG that also serve as PKC-modulating agents [21, 40].

 Of interest is the differential targeting of PKC isoforms by phorbol esters versus certain synthetic or natural DAG analogue lactones. For example, the induction of latent HIV-1 is mediated by the sequential action of PKC *α* and PKC *θ* isoforms in PMA or prostratin-treated T cells [49] while bryostatin-1 treatment accomplishes reactivation through PKC *α* and PKC *δ* [21].

**Figure 2:**
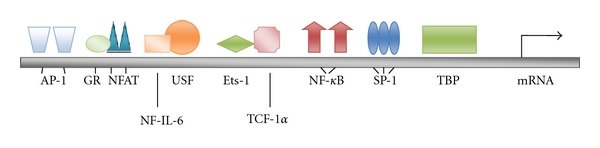
Critical transcription factor binding sites within the HIV-1 long terminal repeat (LTR) that facilitate upregulation of latent HIV-1.

The outcome of PKC isoform activation or modulation by phorbol esters and structurally related agents occurs downstream via transduction through at least the ERK1/ERK2 mitogen-activated protein kinase pathway [50]. As shown in Figure 1, this pathway stimulates IKK-dependent phosphorylation and degradation of I*κ*B*α*, leading to activation of NF-*κ*B [50, 51]. As shown in Figure 2, free NF-*κ*B is then competent for translocation and binding to sites in the enhancer region of the HIV-1 long terminal repeat (LTR). The binding of NF-*κ*B to the LTR is required for high-level transcription of HIV-1 RNA and upregulation in the expression of latent reservoir virus. NF-*κ*B upregulation of latent proviral DNA in reservoir cells is synergized by the stimulation of the activator protein-1 (AP-1). AP-1 activity is stimulated through JNK and mitogen-activated protein kinase signaling pathways which is triggered upstream by PKC action [50, 51]. As shown in Figure 2, the transcription factors AP-1, as well as SP-1, which recognizes GC-rich sequences, have their binding sites located in the HIV-1 LTR enhancer region. It is likely that NF-*κ*B, AP-1, and Sp-1 are cooperative toward accomplishing robust expression of latent viral reservoirs; however, Nf-*κ*B appears to be the central mediator for latent provirus expression [51–53]. An ancillary activity of PKC activation is the phosphorylation of Tat, a virally encoded accessory protein required for transcript elongation to produce full-length HIV-1 RNAs [54].

## 6. Concerns of Modulating PKC Activity

There have been ongoing concerns regarding the clinical use of PKC modulators for HIV-1 reservoir eradication, particularly the phorbol ester family of compounds. As previously stated, phorbol esters activate PKC in multiple cell types raising the issue that systemic effects in treated patients would be broader than necessary given the limited cell types in the latent reservoir necessary to be targeted for eradication.

*In vitro* studies have shown that PMA advances the expression of monocyte and macrophage surface markers in promonocytic and myelocytic leukemia cell lines as well as primary leukemic cells [55]. Indications of cellular differentiation in response to PMA and other phorbol esters include morphological alterations, reduction in the rate of replication, and remodeling of cellular fiber networks leading to either increased cellular adherence to plastic or decreased cell-cell contact [46, 55]. Further, alterations in receptor expression affect responses to external or internal cell signaling.

**Figure 3:**
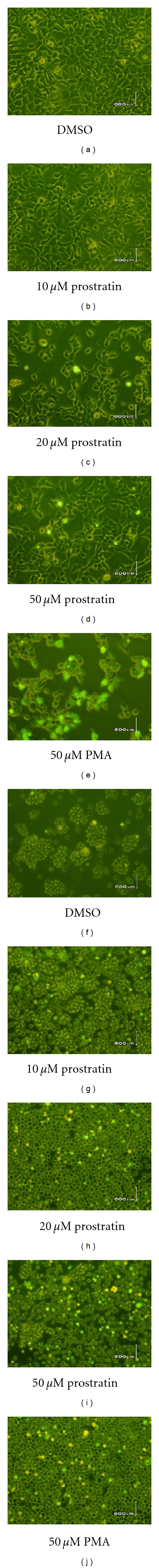
Morphological and apoptotic effects of PMA and prostratin on select cell lines. Top panel: 293 adherent cells. Lower panel: Jurkat cells. Concentrations of prostratin at or below 10 *μ*M, higher than required to upregulate latent HIV-1, do not markedly alter morphogenesis or induce apoptosis. Concentrations >10 *μ*M affect both cellular processes.

 Alterations in cell-cell contact and the induction of apoptosis by phorbol ester exposure can be easily demonstrated and are evident in Figure 3. The cell lines, representing immortalized embryonic kidney 293-A and transformed Jurkat T lymphocytes, were treated with concentrations of prostratin and PMA as indicated in Figure 3. DMSO serves as the negative control. A proapoptotic sensing fluorescent dye was added 1 hour posttreatment. Two phenomena are readily observable. The focal points of fluorescence indicate cells undergoing apoptosis which increase at higher phorbol ester concentrations. As shown in Figure 3, the normally adherent 293 cells detach from the plastic dish and become rounded, while Jurkat T-cell aggregates in suspension disperse and regress from a rounded morphology. While the effects of PKC modulating agents on cells have been well studied, the consequences of PKC modulation on tissues and organ systems have not. Our recent observations on modulating PKC activity in zebrafish indicate that the concentrations of phorbol esters, which readily upregulate latent HIV-1 transcription, do not induce developmental or lethal effects in zebrafish embryos or early-term larvae [56].

 The overwhelming concern with regard to the administration of certain phorbol esters is tumor promotion [57, 58]. Cellular transformation is a notorious property of PMA which occurs through the sustained action of PKC *α* [58]. Importantly, PMA-related phorbols antagonize PMA-mediated tumor promotion of PMA [59] while DAG lactones appear less likely to mediate cellular transformation as a consequence of their low affinity for PKC *α* and preferentially targeting PKC *θ* [21, 60].

The toxicity profile of PMA, alternatively referred to as 12-*O*-tetradecanoylphorbol-13-acetate (TPA), has been clinically evaluated. Low doses administered to patients resulted in transient fever and mild dyspnea [61]. No renal or hepatic complications were noted although one patient suffered a grand mal seizure upon receiving a platelet transfusion after PMA administration. Collectively, the overt clinical side effects of phorbol ester or macrolide lactone administration to patients include respiratory distress syndrome, hypotension, and other toxicities due to the release of proinflammatory cytokines via nonspecific cellular activation [61, 62]. In one study, myalgia and localized phlebitis were dose-limiting side effects of bryostatin-1 administration [63].

## 7. Are PKC Modulators Serious Candidates for Clinical Use?

**Figure 4:**
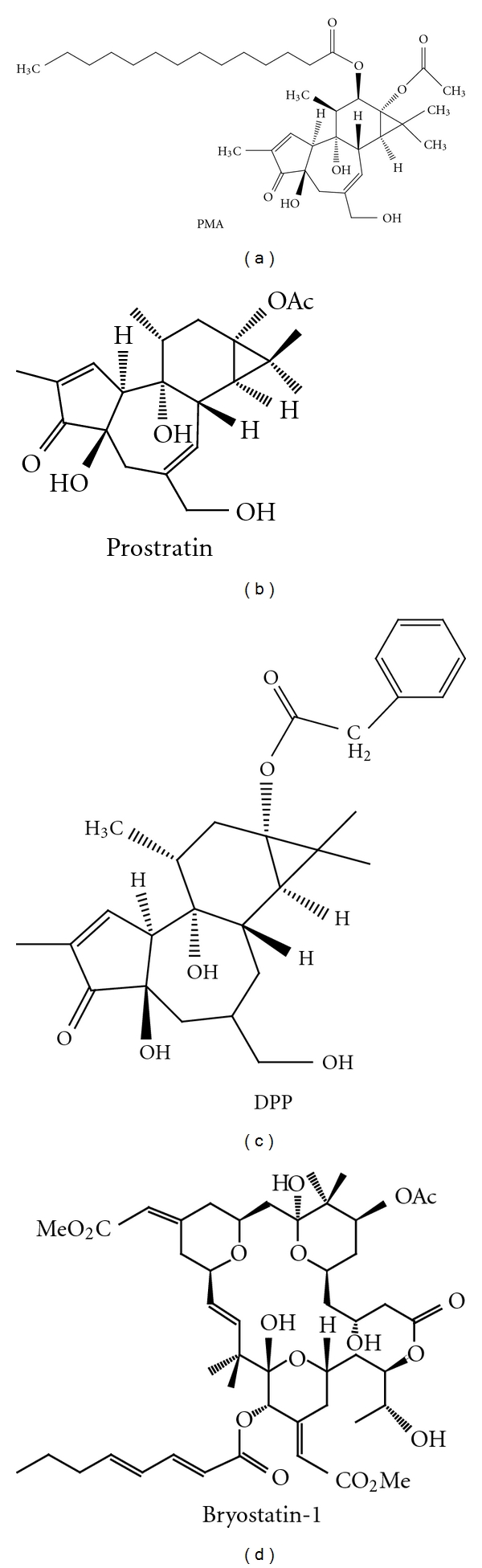
Comparative chemical structures of PKC-modulating agents.

It has been observed that structural variants among the phorbol ester family of agents differ in their potency to upregulate latent viral expression which likely relate to differential PKC isoform affinities for effector targets [20, 40, 59]. *In vitro* concentrations of phorbol esters, required for the upregulation of latent virus in patient primary cells, are in the range of 0.1 *μ*M to 10 *μ*M as single treatment agents [20, 45, 46]. The mechanistic basis for the differences in effective concentrations of agents relates to variations in their chemical structure. As shown in Figure 4, both PMA and DPP have extended hydrophobic side groups which are not present in the base structure of prostratin. These hydrophobic entities exposed outward from the PKC/phorbol complex, likely associate with and retain the complexes at the plasma membrane resulting in sustained PKC action. Such sustained action can accentuate select processes including tumor promotion or apoptosis which are not typical of prostratin treatment relative to PMA and DPP at similar concentrations. On the basis of these observations, it was proposed that the ancillary toxicities of PKC activators could be minimized by altering select reactive groups to present the most favorable clinical index.

 Toward that goal, Hamer et al. synthesized and tested the activity of several DAG analogues that are agonists of the PKC pathway [60]. Among those assessed, LMC03 and LMC07 were less potent than other phorbols at upregulating the proinflammatory cytokine, TNF-*α*. These compounds also had decreased ability to downregulate cell surface expression of CD4 and CXCR4 in A3.01 cells.

 More recently, Márquez et al. demonstrated that phorbol-13-stearate effectively activates latent HIV-1 expression 10-fold more potently than prostratin [40]. Like PMA and prostratin, phorbol-13-stearate stimulates IKK-dependent phosphorylation and degradation of I*κ*B with subsequent activation of NF-*κ*B. Interestingly, phorbol-13-stearate treatment results in the translocation of PKC isotypes *α* and *δ* to different cellular compartments than do prostratin and PMA [40]. Clinical administration of these PKC-modulating derivatives has not yet been reported.

## 8. Conclusion

The ongoing efforts to formulate new lead compounds, to upregulate latent HIV-1 through PKC modulation which bear minimal clinical side effects, appears to be a rational approach toward the eradication of latent HIV reservoirs. Differential PKC isoform targeting may be important since those isotypes that modulate PKC downstream of PKC *α*, exhibit fewer deleterious cellular and tissue side effects. It is unlikely that one singular therapeutic approach will achieve complete reservoir ablation as multiple molecular mechanisms can elicit or maintain HIV-1 latency [64]. The lessons from HAART usage indicate that some form of combination therapy with reservoir eradication agents, as well as multiple dosing, could be of utility. Nonetheless, treatments, which markedly accelerate the process of reservoir decay, should be regarded as a significant advancement toward the goal of curing HIV-1 infection, as well as compelling, given the considerable expense and notable side effects associated with the prolonged administration of HAART.
